# Can Macroevolution Inform Contemporary Extinction Risk?

**DOI:** 10.1111/ele.70171

**Published:** 2025-06-30

**Authors:** Sarah‐Sophie Weil, Sébastien Lavergne, Florian C. Boucher, William L. Allen, Laure Gallien

**Affiliations:** ^1^ Department of Biodiversity, Macroecology & Biogeography University of Göttingen Göttingen Germany; ^2^ Université Grenoble Alpes Université Savoie Mont Blanc, CNRS, LECA Grenoble France; ^3^ Department of Biosciences Swansea University Swansea UK

**Keywords:** conservation, diversification rate, extinction rate, extinction risk, macroevolution, macroevolutionary rates, niche evolution, phylogenetic analysis, speciation rate, traits

## Abstract

Current global changes are driving many species towards extinction, making the early detection of threatened species a priority for efficient conservation actions. However, the threat status of many species remains unknown due to insufficient data on updated distributions, population sizes and population trends and using ecological indicator traits, such as range size, is not always straightforward. Recent advances suggest that macroevolutionary indicators (rates of extinction, net diversification or niche evolution) could provide novel insights into extinction risk based on the assumption that macroevolutionary rates can serve as proxies for extinction‐promoting traits (small range size, narrow niche breadth or low evolutionary potential). However, this assumption has not yet been sufficiently investigated to use this approach. Here, we assess current understanding of the assumptions underlying the relationship between macroevolutionary indices and contemporary extinction risk. We find that only past extinction rates can be reliable predictors of current extinction risk due to their correlation with inherited extinction‐promoting traits. Assumptions underlying relationships between current extinction risk and diversification and niche evolution rates vary by taxon or ecological conditions, and require further investigation through targeted studies. When underlying assumptions are validated, macroevolutionary indicators could be promising tools complementing trait‐based approaches in identifying inherent extinction risk.

## Graphical abstract

We investigate the underlying assumptions of the idea that macroevolutionary indicators for large groups of species, such as rates of extinction, diversification or niche evolution, can inform species' current and future extinction risk status. We find that only past extinction rates can be reliable predictors of current extinction risk as the same traits that are related to past extinctions are also related to current extinction risk. Diversification and niche evolution rates, on the other hand, are currently unlikely to be generally useful predictors because underlying assumptions were only met in specific cases (e.g., restricted geographic space) or hard evidence is only starting to emerge. Before using macroevolutionary indicators to assess species' intrinsic vulnerability or guide data collection priorities, future studies must first verify the validity of underlying assumptions, recognising that final conservation decisions will require additional supporting data.

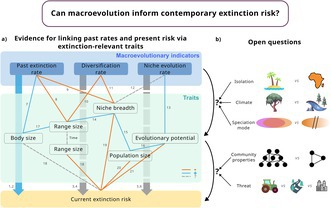

## 1 Introduction

Current global changes are driving many species to extinction, making the identification of species that are and will be at high extinction risk a key priority to take efficient conservation actions (IPBES 2019). However, data for detailed risk assessments, for example, trends of species' population sizes, are not readily available for many species. For example, ca. 15% of all reptile species evaluated by the International Union for Conservation of Nature (IUCN) are classified as data deficient, and ca. 83% of flowering plants have not been evaluated at all (IUCN 2024).

In recent decades, conservation science has significantly progressed in identifying threatened species by using indicators of extinction risk inspired by approaches from population ecology, landscape genetics and macroecology (e.g., R. Huang et al. 2020; Pearson et al. 2014; Sakai et al. 2002; Santini et al. 2021). It is now well established that species' extinction risk generally (but not always) increases with small range, narrow niche, low fecundity and/or long generation time (e.g., Chichorro et al. 2019, 2022; Forsman 2014; Harnik, Simpson, and Payne 2012; Lucas et al. 2024). These indicators have led to the development of trait‐based predictive models to estimate the risk of extinction for species currently unassessed by the IUCN (Bachman et al. 2024; Bland et al. 2015; González‐del‐Pliego et al. 2019; Kopf et al. 2017; Santini et al. 2019). However, relationships between individual ecological traits and extinction risk depend on the taxonomic group, the geographic context and the type of threat species face (Chichorro et al. 2019; Fritz et al. 2009; Gonzalez‐Suarez et al. 2013), and measuring many of these ecological traits is cost‐intensive because it requires extensive fieldwork. Predicting which species are most likely to be or become threatened therefore remains challenging.

Recent studies have investigated correlations between macroevolutionary rates inferred from phylogenies, such as rates of extinction, diversification or niche evolution and current extinction risk (e.g., Davies et al. 2011; Greenberg et al. 2021; Lavergne et al. 2013; Tanentzap et al. 2020). For instance, high past diversification rates were linked to increased current IUCN extinction risk in plants (e.g., Greenberg et al. 2021), and slow rates of niche evolution were linked to recent demographic declines in birds (Lavergne et al. 2013). Based on these correlations, authors have suggested that past macroevolutionary rates could be used to estimate the current threat status of unassessed species or define certain clades as priorities for conservation (e.g., Tanentzap et al. 2020; Ye et al. 2024), despite the differences in timescales and possible differences in external drivers influencing extinction risk (see also section 5. Synthesis and Recommendations).

**FIGURE 1:**
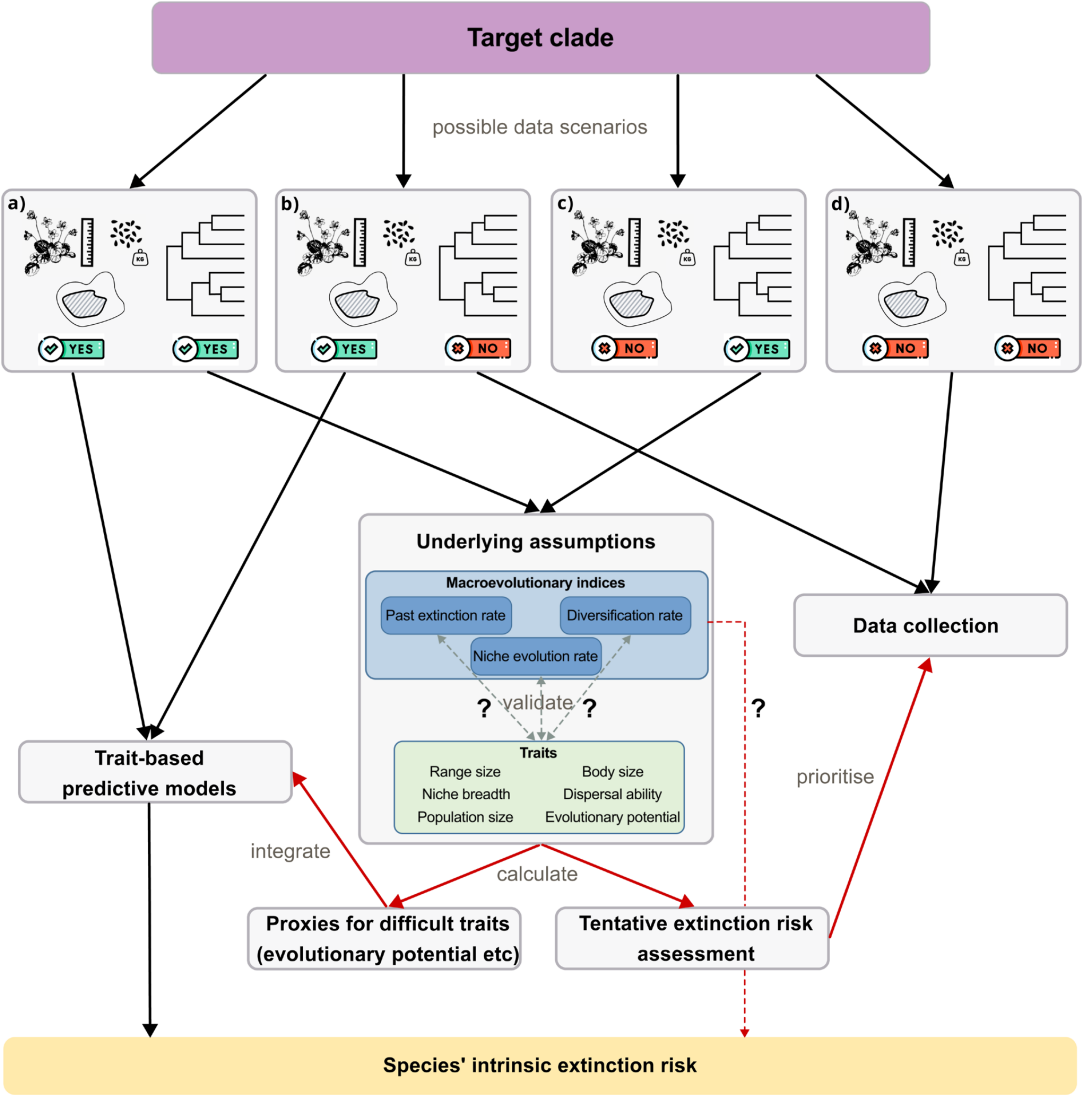
Framework on how macroevolutionary indicators can be integrated in the assessment of species' intrinsic extinction risk, depending on different scenarios of data availability (a–d). Macroevolutionary indicators could be integrated into trait‐based predictive models of extinction risk as proxies for traits that are difficult to measure (e.g., evolutionary potential), or they could be used for a tentative extinction risk assessment which could help prioritise further data collection efforts. This approach only works if underlying assumptions between macroevolutionary indicators and extinction‐relevant traits, such as range size, niche breadth or evolutionary potential, are validated (possibly in related clades or similar geographic contexts). If the assumptions are not validated, the steps indicated by red arrows are not possible, decreasing the pool of available information for extinction risk assessments. Icons: flaticon.com.

This approach holds a lot of promise due to recent advances towards estimating the evolutionary history of life at large scale. If macroevolutionary indicators can reliably predict species' extinction risk, they could rapidly expand the number of species covered by extinction risk assessments because extinction‐relevant information could become available for all species with reliable phylogenetic information (Wilder et al. 2023). Currently, genetic information is, for instance, available for 7195 of described squamate species (i.e., ca. 76%, Oskyrko et al. 2024). Not all of these are included in the commonly used squamate phylogeny (Tonini et al. 2016), but they could potentially be included in the future. Yet, 16% of these species are declared data deficient or have not been assessed by the IUCN, and 28% (i.e., 1986 species) have only size‐related or morphological trait data (Oskyrko et al. 2024, disregarding range based data, such as biogeographic region or climate data). If macroevolutionary indicators allowed a good approximation of extinction risk, or extinction‐relevant traits such as adaptive potential (see next paragraph), this would increase (1) our ability to include more information in estimates of species' vulnerability to global changes (i.e., intrinsic extinction risk) and/or (2) allow us to prioritise species for which further data collection efforts are crucial (i.e., horizon‐scanning approaches). However, the validity of this framework hinges on underlying mechanistic connections between macroevolutionary indicators and current extinction risk (Figure 1).

It is known that species' current extinction risk is directly related to traits that define their vulnerability to environmental changes, such as narrow niche breadth or small range and population sizes, and their adaptive capacity (hereafter called extinction‐promoting traits, Box 1). Thus, if macroevolutionary indicators are indeed mechanistically linked to extinction risk, this relationship should also be mediated by traits or trait combinations. That is, if evolutionary rates measured over historical timescales reflect processes that are still happening today (such as demographic trends, trait or niche evolution), then heritable traits should underlie these processes and macroevolutionary indicators could be useful proxies for species' contemporary population and range dynamics (Dietl and Flessa 2011; Fritz et al. 2013; Pimiento and Antonelli 2022) or species' ability to evolve their niches (e.g., Edwards and Donoghue 2013; Nogués‐Bravo et al. 2018; Román‐Palacios and Wiens 2020). For instance, a fast rate of niche evolution in a lineage could indicate that extant species hold high capacities to adapt to new environmental conditions, which would lead to decreased extinction risk (e.g., Lavergne et al. 2013; Salamin et al. 2010). Yet, empirical tests of how extinction‐promoting traits relate to evolutionary rates are scattered and inconsistent. This prevents us from going forward in using macroevolutionary indicators to inform current extinction risk.

BOX 1 **Correlates of Contemporary Extinction Risk.**

Species' current extinction risk is not random but depends on species' characteristics. Three main features have been linked to extinction risk: the breadth of species' niches, their capacity to adapt to new conditions and their life history traits.

Regarding species' niches, the risk of extinction tends to increase with specialised dietary and habitat preferences (Chichorro et al. 2022; Colles et al. 2009; Devictor et al. 2008; Ducatez et al. 2014; Harnik, Simpson, and Payne 2012; Olden et al. 2008; Smits 2015). Similarly, extinction risk increases for species with limited geographic distribution (Koh et al. 2004; Lavergne et al. 2005, 2006; Matthies et al. 2004; Sakai et al. 2002), and small population size (Caughley 1994; Pimm et al. 2007; Purvis, Jones, and Mace 2000; Wilder et al. 2023). This is because small populations face a number of problems, including increased vulnerability to environmental stochasticity and inbreeding depression (Ovaskainen and Meerson 2010). As the characteristics linked to extinction risk often covary, additive effects can emerge: highly specialised species are often geographically restricted (Brown 1995; E. Huang et al. 2021; Slatyer et al. 2013, but see Fargallo et al. 2022) and have small population sizes (Gaston 1994). Small‐ranged specialists are hence generally considered to be the most prone to extinction (Malanoski et al. 2024).

Further, the risk of extinction has been linked to species' adaptive capacity, that is, their ability to respond to environmental changes through behavioural flexibility, evolution and/or dispersal (Beever et al. 2017; Foden et al. 2019; Forester et al. 2022). Species with low evolutionary potential, for example due to low genetic diversity, are less likely to adapt to novel conditions (de Villemereuil et al. 2019; Frankham et al. 1999; Hoffmann et al. 2017; Kardos et al. 2021). Conversely, high evolutionary potential can prevent extinctions by favouring evolutionary rescue following adverse environmental change (Gonzalez et al. 2013; Vander Wal et al. 2013). Species with high dispersal potential also have a lower extinction risk because they can track spatially shifting favourable conditions (Román‐Palacios and Wiens 2020; but see Thompson and Fronhofer 2019). Conversely, species with low dispersal ability are expected to be at higher extinction risk. In birds, for instance, flightless species went disproportionately extinct during the late Pleistocene and Holocene (Sayol et al. 2020). Both species' evolutionary potential and dispersal abilities are not independent from the extinction correlates mentioned previously; for example, species with good dispersal abilities tend to have larger ranges and species with large populations tend to have higher evolutionary potential (Alzate and Onstein 2022; Habel and Schmitt 2012).

Finally, a large number of studies have focused on the relationships between extinction risk and traits such as body size and life history. Large body size is often associated with increased extinction risk, but has been shown to depend on threat type (reviewed in Chichorro et al. 2019), while the relationships between life history traits and extinction risk are more variable, with no general pattern emerging (Bennett and Owens 1997; Cardillo et al. 2005; Harnik, Simpson, and Payne 2012; Hutchings et al. 2012; Kemp and Hadly 2015; Reynolds 2003).

To summarise, species with the following characteristics are generally expected to be at high risk of extinction: narrow niche breadth, small range and population sizes, low evolutionary potential, low dispersal potential and large body size.

The idea of using macroevolutionary indicators to inform conservation is promising; however, there is a risk that this approach will be used inappropriately if underlying assumptions are not considered. In particular, it is rarely tested that macroevolutionary rates correlate with extinction‐promoting characteristics (hereafter ‘underlying assumption’), which calls the validity of the direct link between macroevolutionary rates and contemporary extinction risk into question. Here, we clarify the state of knowledge on the mechanisms that link macroevolutionary rates and current extinction risk so that future work can pursue the integration of macroevolution in conservation science efficiently. In the following sections, we first summarise results from studies that link macroevolutionary rates directly to present‐day extinction risk, and then we review scientific evidence to see if and when the underlying assumptions hold, separately for extinction rates (section 2), diversification rates (section 3) and niche evolution rates (section 4). Knowing in which cases the relationships between macroevolutionary rates and extinction‐promoting traits are supported is crucial to using macroevolutionary indicators to their full potential in a conservation context and to ultimately answer the question: Can we use information from macroevolutionary studies to help understand contemporary extinction risk, and if so, under what conditions?

## 2 Past Extinction Rate as Proxy for Current Extinction Risk

Studying past extinctions may help us better understand causes of extinction and which species are especially under threat (Condamine et al. 2013; Finnegan et al. 2015; Harnik, Lotze, et al. 2012; McKinney 1997), even though rates of extinction today are estimated 1000 times higher than historical background rates (Pimm et al. 2014). Few studies have explicitly tested the relationship between past extinction rates and current extinction risk, but those that did generally found a positive relationship between them: high past extinction rates are related to elevated extinction risk today (Table 1). For instance, in corals, those genera that suffered regional extinctions during the Plio‐Pleistocene are also at elevated risk today (estimated through a trait‐based resilience score, van Woesik et al. 2012). In bivalve families, fossil‐based extinction rates of the Mesozoic were correlated with those of the Cenozoic, and extinction rates of closely related families were more similar than expected by chance (Roy et al. 2009).

**TABLE 1:** Studies quantifying the link between macroevolutionary indicators (extinction, diversification and niche evolution rates) and contemporary extinction risk at different taxonomic levels identified following comprehensive literature search.

Macroevolutionary indicators	Relationship with extinction risk		
	Positive	Negative	Not significant
Extinction rates	Corals (van Woesik et al. 2012), bivalves (Roy et al. 2009)		
Diversification rates	Plants (Davies et al. 2011; Fu et al. 2022; Schmidt et al. 2021; Tanentzap et al. 2020), cetaceans (Condamine et al. 2013), amphibians (Greenberg and Mooers 2017), tetrapods (Greenberg et al. 2021)	Angiosperms (within biodiversity hotspots: Fu et al. 2022; Ye et al. 2024; globally: Vamosi and Wilson 2008, ^a^), marsupials (Johnson et al. 2002, ^a^), primates (at species‐level; Verde Arregoitia et al. 2013, ^b^), mammals (Purvis, Agapow, et al. 2000, ^a^), birds (Purvis, Agapow, et al. 2000, ^a^; Von Euler 2001, ^a^), turtles and crocodiles (Colston et al. 2020, ^b^)	Endemic angiosperms of China (Yu et al. 2022), mammals (at genus‐level; Verde Arregoitia et al. 2013; Verde Arregoitia et al. 2013, ^b^), birds (Jetz et al. 2014, ^b^), squamates (Tonini et al. 2016, ^b^)
Niche evolution		European birds (Lavergne et al. 2013)	Terrestrial vertebrates (Rolland et al. 2020)

^a^ Results based on loss of phylogenetic diversity (higher loss of PD than expected by chance is assumed to indicate low diversification rates being related to high extinction risk).

^b^ Results based evolutionary distinctiveness (phylogenetically isolated species being at higher extinction risk indicates low diversification rates being related to high extinction risk).

**FIGURE 2:**
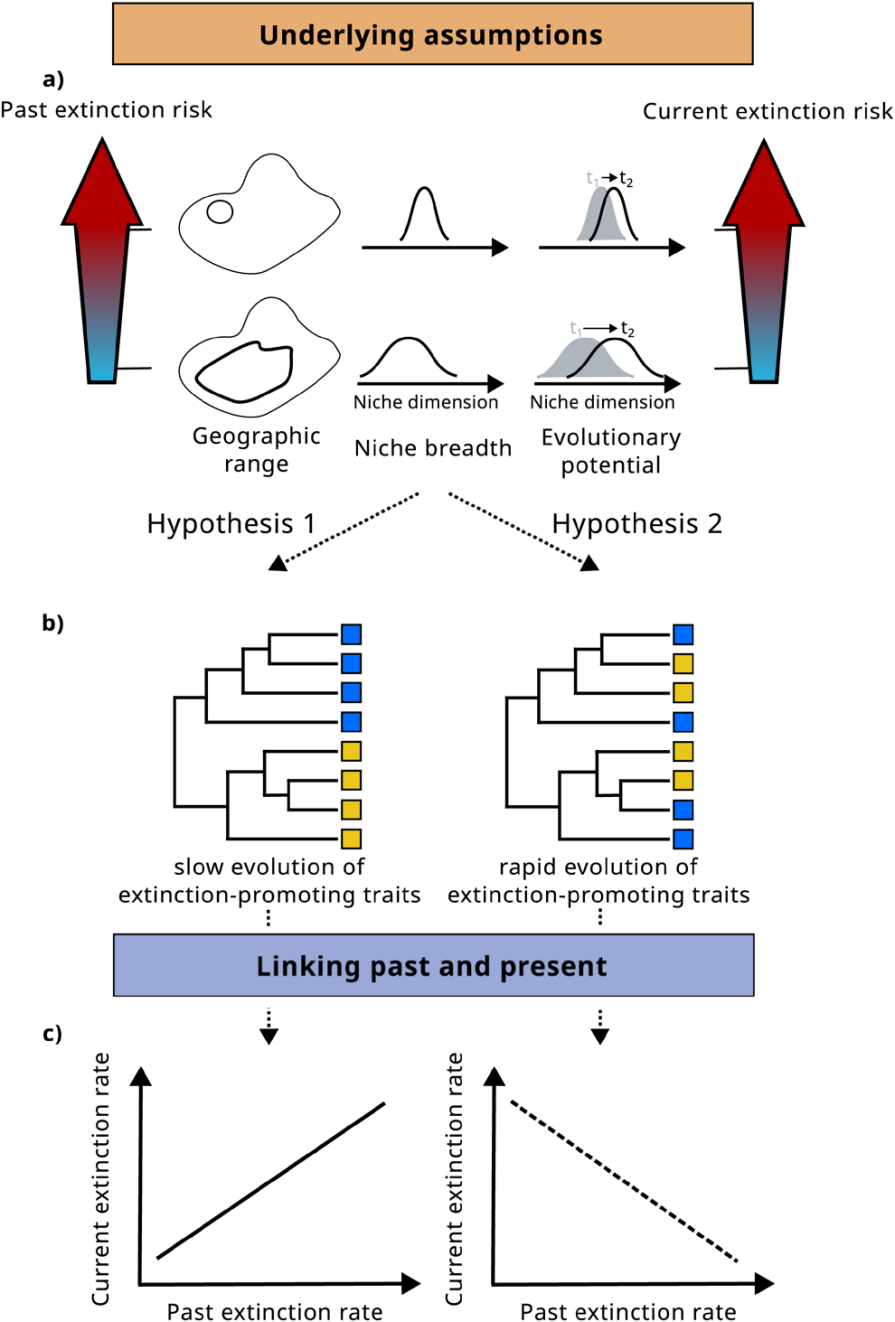
Hypotheses linking past extinction rate and current extinction risk. If past and current extinction processes are linked to the same traits (panel a) and evolution of extinction‐promoting traits is slow (panel b), that is, descendants are likely to have similar trait states as their ancestors, then species descending from lineages with high past extinction rates are at high risk of extinction today (panel c). If evolution in extinction‐promoting traits is rapid, then species descending from lineages with high extinction rates are expected to be at low extinction risk today.

There are two contrasting hypotheses of how past extinction rates may be linked to present‐day extinction risk (Figure 2): (Hypothesis 1) Rates of extinction vary among clades due to lineage‐specific traits and characteristics, such as range size or degree of specialisation. Species descending from lineages with high extinction rates have inherited the traits of their ancestors, and may hence be especially at risk of extinction today (e.g., Roy et al. 2009 and references therein). (Hypothesis 2) Alternatively, species still surviving from lineages with high extinction rates may be less at risk of extinction because they possess the features that made them particularly resistant to past extinctions (D. Jablonski 1994). Both hypotheses thus rely on a number of assumptions. Hypothesis 1 assumes that the same traits that are related to present‐day extinction risk were also linked to past extinctions, and that these traits have evolved slowly, that is, extinct and extant species have similar traits. Hypothesis 2 agrees with the first part of hypothesis 1 but assumes in contrast that extinction‐promoting traits have evolved rapidly, that is, extant species have different traits than extinct species and extinction‐promoting traits were counter‐selected. To identify which of the contrasting hypotheses is best supported, we investigate in the following section each assumption underlying the link between past and current extinction risk.

### 2.1 The Evidence for Underlying Assumptions—Linking Past Extinction to Traits Related to Current Extinction

Species' characteristics linked to past and current extinctions are remarkably similar, shown for example in amphibians where species' current IUCN extinction risk categories were well‐predicted with a trait‐based model trained on extinct species (Tietje and Rödel 2018; see also McKinney 1997). Small geographic range size is a strong predictor of past extinction risk (Guinot and Condamine 2023; Harnik, Lotze, et al. 2012; Kiessling and Kocsis 2016; Malanoski et al. 2024; Payne and Finnegan 2007; Purvis, Jones, and Mace 2000), as is large body size (Boyer 2010; Kemp and Hadly 2015). The relationship between large body size and pre‐human extinctions has been explained by the negative relationships between body size and reproductive rates and population density (Peters 1983 in Kemp and Hadly 2015). Ecological specialisation was a determinant of extinctions in marine mammals and marine invertebrates over the last 500 million years (Harnik, Simpson, and Payne 2012; Kammer et al. 1998) and slow life histories in terrestrial mammals during the Quaternary (N. G. Jablonski et al. 2000; Johnson 2002). Endemism, particularly island endemicity, was related to increased extinction risk in birds during the Holocene (Boyer 2010; Duncan et al. 2013; Fromm and Meiri 2021).

In order to use past extinction rates as a proxy for current extinction risk, it is necessary to know whether descendants of lineages with extinction‐promoting characteristics hold similar traits (i.e., extinct and extant species). While heritability in individual traits, such as body size is undisputed (reviewed in Freckleton et al. 2002), heritability of emergent species‐level traits, such as range size or niche breadth, is more contested (Borregaard et al. 2012; D. Jablonski 1987, 2008). In the case of range size, several studies show that closely related species tend to have more similar range sizes, indicating a slow rate of evolution. However, the rate of evolution of range size varies greatly across clades (e.g., mammals: Cardillo 2015; Pie and Meyer 2017; squamates: Pie and Meyer 2017; birds: Waldron 2007; tropical plants: Loza et al. 2017; ammonites: Zacaï et al. 2017). In contrast to range size, niche breadth similarity in closely related species is less frequent, often depending on geographic and taxonomic context (Hof et al. 2010; Kamilar and Cooper 2013; Losos 2008).

Niche breadth evolution has been shown to vary across different settings. Rapid generalist‐to‐specialist transitions have been documented in clades that include both generalist species and nested adaptive radiations (where a generalist ancestral species diversified into specialised species), but also in clades without adaptive radiations (e.g., Jacquemyn et al. 2024; Knouft et al. 2006; Losos 2008). Specialist‐to‐generalist transitions tend to be slow as specialist ancestors tend to have specialist descendants (Allen et al. 2020; Brändle et al. 2002). However, there are numerous counterexamples showing that specialists can evolve rapidly into generalists (reviewed in Colles et al. 2009), and even that specialist‐to‐generalist biases exist (i.e., higher probabilities of specialists evolving into generalists than the other way around, Sexton et al. 2017). As concluded by Schluter (2000), the direction of evolution of niche breadth may just be unpredictable.

Altogether, there is some empirical support for hypothesis 1, that the same traits are related to present‐day and past extinctions (see also Smits and Finnegan 2019), and that most of these traits evolve relatively slowly, such as body size and range size, suggesting that closely related species tend to share similar extinction risks. Heritability of extinction is also supported by the fact that models where extinction rates are phylogenetically structured approximate the shape of observed phylogenies well (Rabosky 2009). However, there are strong variations in the rate of trait evolution among clades and it appears to be difficult to predict in some extinction‐promoting traits (i.e., specialisation). Therefore, past extinction rates can be useful predictors for current extinction risk once the tempo and mode of extinction‐promoting trait evolution has been assessed. It should be noted that, if possible, it is important to account for the traits of extinct lineages when assessing the evolution of extinction‐promoting traits for this purpose. If all surviving species within a clade possess traits that differ from their extinct sister species, this divergence could be underestimated. So far, there is no evidence for hypothesis 2 as the direct relationships between past and current extinction risk were found to be positive.

## 3 Past Diversification Rate as Proxy for Current Extinction Risk

For many clades, past extinction rates cannot be reliably estimated because the fossil record is incomplete and biased towards some taxa. At the same time, extinction is difficult to infer from phylogenetic trees (Nee 2006; Rabosky 2010) because the underlying diversification process (e.g., rates of speciation and extinction across lineages) needs to be correctly identified, which can be challenging (Louca and Pennell 2020; Morlon et al. 2011, 2024). Therefore, studies using phylogenetic comparative methods to link current extinction risk to past rates generally do not investigate direct relationships between current and past extinction. Instead, they analyse the relationship between current extinction risk and net diversification rates (the difference between speciation and extinction), which are easier to estimate reliably from phylogenetic trees based on branching times or even only on data of extant species richness and clade age. Substitutes of diversification rates based on phylogenetic diversity (the sum of branch lengths connecting species on a phylogenetic tree, e.g., Heard and Mooers 2000; Purvis 2008) or evolutionary distinctiveness (a measure of how isolated a species is on a phylogenetic tree relative to other members of its clade, e.g., Tonini et al. 2016) were also used in the literature before the development of models that estimate diversification rates directly.

Previous studies found that the link between the diversification rates of clades and the proportion of endangered species they contain (as categorised by the IUCN) depends on taxonomic and geographic context (Table 1). Relationships were positive in some groups of plants and tetrapods (Condamine et al. 2013; Davies et al. 2011; Fu et al. 2022; Greenberg et al. 2021; Greenberg and Mooers 2017; Schmidt et al. 2021; Tanentzap et al. 2020). In other clades, relationships were predominantly negative (e.g., Fu et al. 2022; Ye et al. 2024). In contrast to this, some studies using phylogenetic diversity as a proxy of diversification rate to study its relationship with extinction risk found that slowly diversifying clades seem to have a particularly high risk of extinction in primates, marsupials, mammals, birds and angiosperms on a global scale (as deduced from higher loss of phylogenetic diversity when threatened species are removed from the phylogeny than expected under random extinction; Johnson et al. 2002; Purvis, Agapow, et al. 2000; Vamosi and Wilson 2008; Von Euler 2001). The effect of estimated diversification rates (using evolutionary distinctiveness as a proxy) on current extinction risk was also negative in small taxonomic groups of reptiles and primates (i.e., phylogenetically isolated species being at higher IUCN extinction risk; Colston et al. 2020; Verde Arregoitia et al. 2013). In other groups, studies have found no relationships between diversification rate and current extinction risk, for example, endemic angiosperms of China, large groups of mammals, birds and squamates (Jetz et al. 2014; Tonini et al. 2016; Verde Arregoitia et al. 2013; Yu et al. 2022).

**FIGURE 3:**
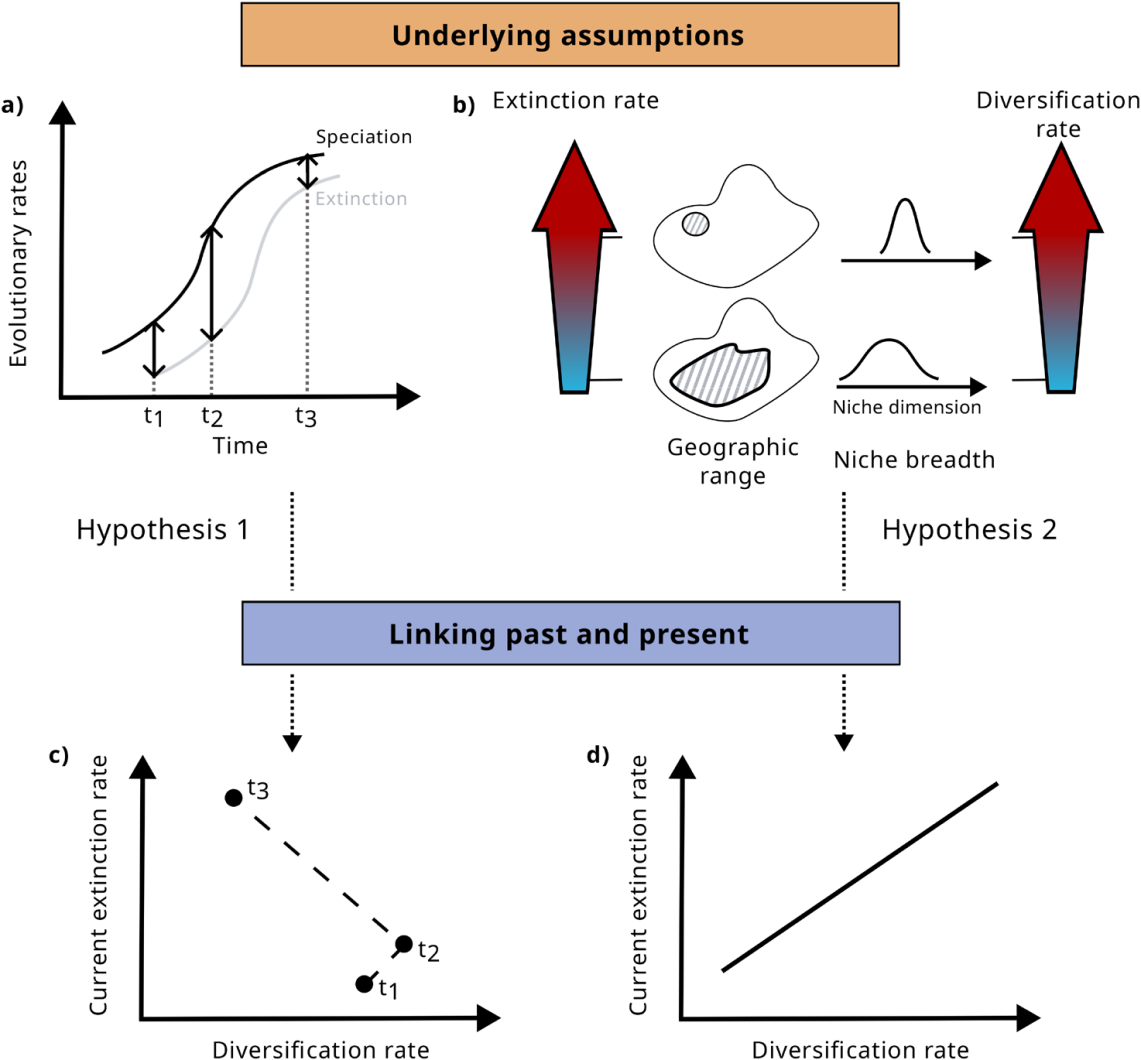
Hypotheses linking diversification rates and current extinction risk. Hypothesis 1 assumes that speciation and extinction rates are correlated but that extinction lags behind speciation (panel a). This can lead to unpredictable behaviour in the relationship between recent diversification rates (the difference between speciation and extinction rate, indicated by the arrows in panel a) and current extinction risk (panel c). Hypothesis 2 assumes that high diversification rates are indicative of extinction‐promoting characteristics, such as small geographic ranges and narrow niches (panel b). This would lead to a positive correlation between diversification and current extinction risk (panel d).

**FIGURE 4:**
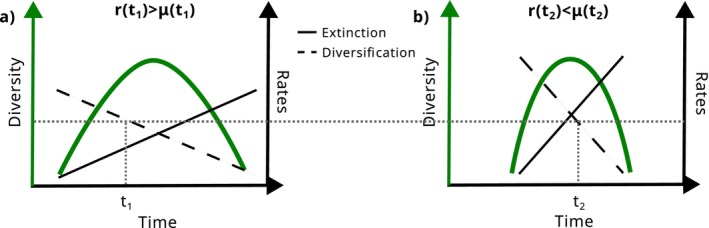
A multitude of scenarios can lead to similar diversification rates. In both panels, a similar diversification rate occurs at different points in the history of the example clades (*t*_1_ and *t*_2_). In (a), the clade is still rising (diversification rate *r* is greater than extinction rate *μ*), whereas in (b), the clade is in decline (*r* is smaller than *μ*).

Since diversification rates are the result of both speciation and extinction, the assumptions underlying their relationship with current extinction risk differ from those between past and current extinction risk (Figure 3; Greenberg et al. 2021; Tanentzap et al. 2020). In particular, it is assumed that (1) extinction and speciation rates are correlated because speciation is facilitated by the same traits that increase extinction risk (e.g., habitat specialisation, low dispersal ability). As extinction necessarily lags behind speciation, lineages with high net diversification rates can thus be either at low or high current extinction risk, depending on the temporal dynamics of the clade (see next section and Figure 4). Diversification rates could also be a useful proxy for current extinction risk if (2) diversification rates were linked to extinction‐promoting characteristics, such as small ranges, and a high degree of specialisation. This has been suggested because the process of speciation itself can increase extinction risk as young species tend to have smaller ranges and population sizes and narrower niche breadths. We discuss each of these underlying assumptions in the next section.

### 3.1 The Evidence for Underlying Assumptions—Linking Diversification and Traits Related to Current Extinction

The first line of underlying assumptions is based on the relationship between past speciation and past extinction. Past speciation and extinction rates are indeed generally correlated based on the paleontological record (Stanley 1990) and traits facilitating speciation are the same that are linked to increased current extinction risk (see review in Jablonski 2008 and Greenberg and Mooers 2017). At the genus level, increases and decreases in clade diversity has been shown to be symmetrical in terrestrial mammals and marine invertebrates (Foote 2007; Quental and Marshall 2013). Clades with high initial diversification rates where speciation far exceeds extinction should consequently also show the steepest declines, that is, the highest extinction rates or highest proportion of vulnerable species, once a threshold has been reached. This can be explained by diversity‐dependent rates (decreasing speciation and increasing extinction rates with increasing clade diversity) overshadowed by a decay of equilibrium diversity due to a deteriorating environment (Quental and Marshall 2013). Extinction rates were also found to increase with time in marine mammals, butterflies and plants in a recent analysis of paleodiversity dynamics based on molecular phylogenies, whereas speciation rates stayed constant (Mazet et al. 2023).

While past speciation and extinction are correlated at large taxonomic levels and there is evidence for high speciation begetting high extinction, the temporal scale needs to be considered. Extinction necessarily follows speciation, but with only the diversification rate of a clade estimated from a phylogenetic tree it is almost impossible to know if the clade is still in the rising phase or already declining (Figure 4). This is because a multitude of different scenarios can lead to the same net diversification rate, and additional data, for example, paleontological data, a priori hypotheses or statistical regularisation techniques are necessary to solve identifiability issues (Louca and Pennell 2020; Morlon et al. 2022). Depending on clade‐specific dynamics, high past diversification rates could be linked to both increased or decreased current extinction risk. In addition, the correlation between speciation and extinction is modified by subclade variation of temporal diversity dynamics. Dynamics at subclade‐level do not necessarily reflect dynamics of entire clades (Mazet et al. 2023).

Independent from the complex temporal diversification dynamics of clades, diversification rates could be used to predict current extinction risk if there are consistent relationships between diversification rates and extinction‐promoting characteristics such as specialisation and small range size.

Specialists tend to have higher diversification rates than generalists. This was found when considering (i) biome specialisation in ruminants, squirrels and butterflies (Cantalapiedra et al. 2011; Gamboa et al. 2022; Menéndez et al. 2021), (ii) host‐plant specialisation in butterflies (Hardy and Otto 2014) and (iii) climatic niche specialisation in amphibian, mammal and bird species (Rolland and Salamin 2016). Many specialists can emerge rapidly through speciation in a clade if there is room for geographical or ecological speciation (Futuyma and Moreno 1988; Hughes and Atchison 2015; Hughes and Eastwood 2006; Vrba 1987). Generalists can also have high diversification rates if they have large ranges, which are more likely to be fragmented by geographical barriers leading to allopatric or peripatric speciation, after which species generally occupy narrower niches than parent species (Castiglione et al. 2017; Rolland and Salamin 2016; Sexton et al. 2017).

Turning towards the relationship between diversification rates and range size, studies found both negative and positive correlations, depending on the taxonomic group. Negative relationships were found in many taxa of plants and tetrapods (Greenberg et al. 2021; Greenberg and Mooers 2017; Leão et al. 2020) while positive relationships were found in turtles, crocodiles, primates, Australian mammals and Chinese angiosperms (Cardillo et al. 2003; Colston et al. 2020; Redding et al. 2010; Ye et al. 2024). Discrepancies in results could be the consequence of these studies not using process‐based models to explicitly model the effect of range size on diversification, instead using correlations between current ranges and tip estimates of diversification rates (like the diversification rate (DR) metric; Jetz et al. 2012). These correlative relationships strongly depend on post‐speciation changes in range size, which can vary substantially between and among clades (Miller 1997; Taylor and Gotelli 1994; Webb and Gaston 2000; Willis 1922). A recent study on mammals that used process‐based models to account for cladogenetic changes in range size found that large‐ranged species had higher diversification rates but that they were more likely to produce at least one small‐ranged daughter species (Smyčka et al. 2023; see also Castiglione et al. 2017). This is consistent with theory whereby large ranges generally increase the variability of environmental conditions a species encounters, which may increase opportunities for local adaptation and hence probabilities of diversification (Darwin 1859; Gaston and Chown 1999). Smyčka et al. (2023) also showed that the correlative relationship between the DR metric and range size was significantly negative, despite large‐ranged species diversifying faster, because large‐ranged species split into small‐ranged species in the speciation process. However, they highlight several clades that are an exception to this rule, where small‐ranged species diversify faster than large‐ranged ones; often cases of radiations in complex geographical settings such as oceanic or continental island settings.

In summary, it is difficult to generalise the relationship between diversification rates and current extinction‐promoting characteristics: while high diversification rates seem to be linked to specialisation, the relationship between diversification and range size generally depends on the evolution of the range post‐speciation. However, in particular geographical settings with many internal barriers (e.g., oceanic archipelagos, lake systems or mountain ranges) high past diversification rates are likely to indicate small‐ranged and specialised species with a high current extinction risk.

## 4 Niche Evolution Rates as Proxy for Current Extinction Risk

Species' evolutionary potential, that is, the ‘capacity to evolve genetically based changes that increase fitness under changing conditions’ (Forester et al. 2022), is an emerging focus of conservation ecology (Catullo et al. 2015; Gonzalez et al. 2013; Hoffmann et al. 2015; Nogués‐Bravo et al. 2018; Olivieri et al. 2016). This understanding is crucial as it helps predict how species may adapt to rapid environmental changes driven by climate change and human activity. As direct evolutionary potential data is scarce, some authors (e.g., Gudde and Venditti 2016; Lavergne et al. 2013; Salamin et al. 2010) have suggested that rate of past niche evolution could be a suitable proxy. Niche evolution rate, which measures how much variance in niches among species in a given clade is accumulated per unit time (Felsenstein 1985; O'Meara 2012), can be estimated from species‐level phylogenies and extant species' niches. Although niche evolution rate is more difficult to measure than other macroevolutionary indicators or traits directly linked to extinction risk (e.g., specialisation), it is still easier to measure than evolutionary potential.

The association between past rates of niche evolution and species' current extinction risk has received mixed empirical support (Table 1). It has been confirmed for European birds, where slow evolution of the climatic and habitat niche was related to recent demographic declines (Lavergne et al. 2013). However, a large‐scale study covering more than 11,000 terrestrial vertebrates did not find any consistent relationship between niche evolution rates and current IUCN threat status (Rolland et al. 2020).

**FIGURE 5:**
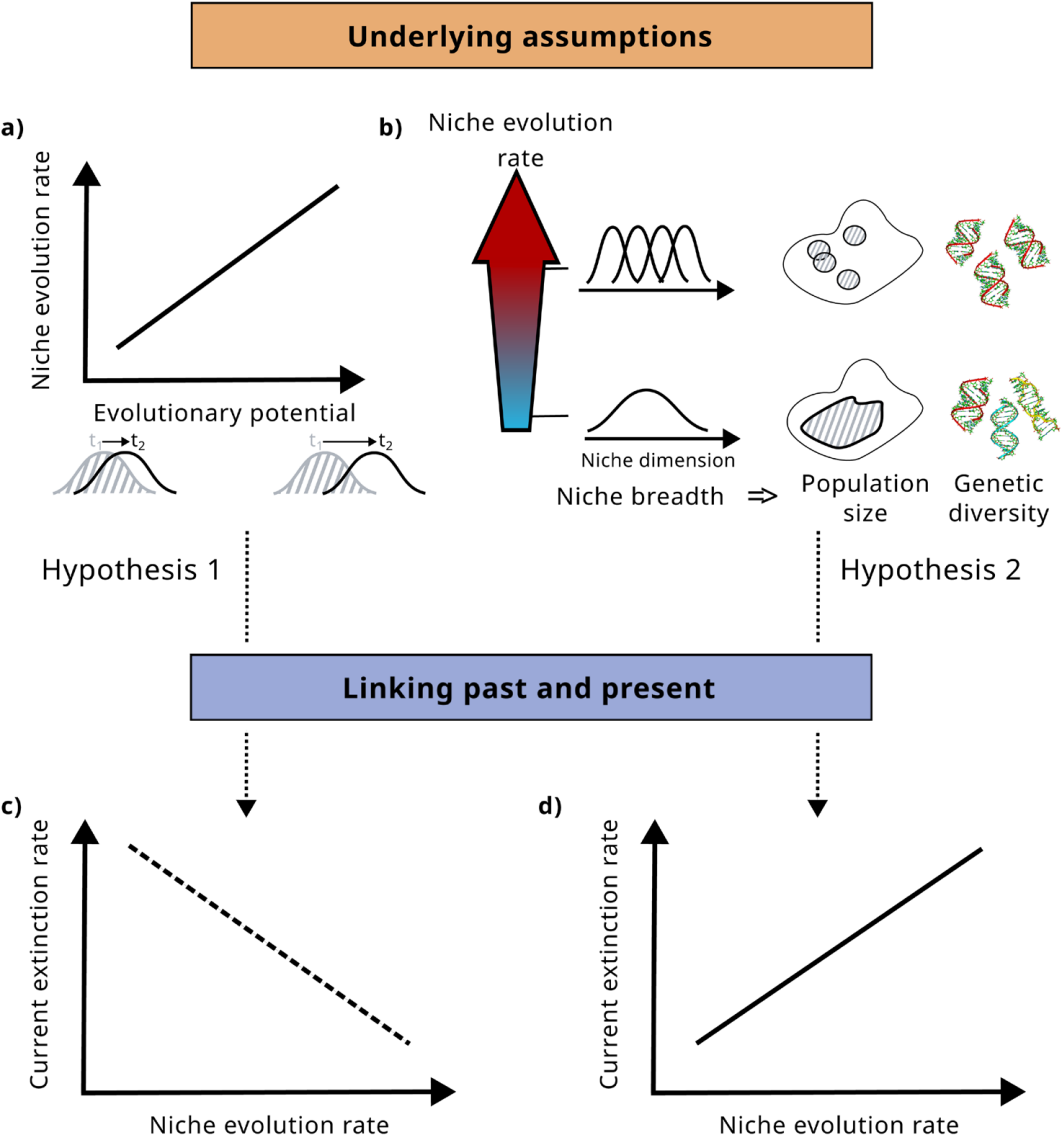
Hypotheses linking niche evolution rates and current extinction risk. If high niche evolution rates are indicative of high evolutionary potential (panel 1), species descending from lineages with high niche evolution rates are likely to be at low current extinction risk (hypothesis 1, panel c). Conversely, if high niche evolution rates are indicative of extinction‐promoting characteristics, such as narrow niche breadth which is often correlated with small population sizes and low genetic diversity (panel b), then species descending from lineages with high niche evolution rates are likely to be at high current extinction risk (hypothesis 2, panel d).

There are two possible ways past niche evolution can be related to current extinction risk (Figure 5): (1) Rates of niche evolution are faster in species with higher evolutionary potential, which should give them an adaptive advantage in the context of current environmental changes (Lavergne et al. 2013; Salamin et al. 2010). (2) Alternatively, within a geographically restricted area where physical barriers to gene flow are likely absent, high rates of niche evolution are more likely to be adaptive than non‐adaptive and hence to lead to more specialisation (Gudde and Venditti 2016) which increases present‐day extinction risk (see above). This effect could be reinforced by the associations between degree of specialisation, population size and genetic diversity, implying that specialist species with isolated populations have low genetic diversity (Habel and Schmitt 2012) and lower evolutionary potential, increasing present‐day extinction risk even further.

### 4.1 The Evidence for Underlying Assumptions—Linking Niche Evolution and Traits Related to Current Extinction

The first hypothesis described above linking niche evolution rates and current extinction risk relies on the positive relationship between niche evolution rates and evolutionary potential. Holstad et al. (2024) compared the relationship between degree of niche evolution and evolutionary potential in contemporary and fossil datasets and found that trait divergence (measured as trait divergence at population and species level in the present and changes in trait means across time in the past) was positively correlated with evolvability (measured as within‐population mean‐scaled additive genetic variance in the present and mean‐scaled within‐sample phenotypic variance in the past).

The second hypothesis linking niche evolution rates and current extinction risk relies on the relationship between niche evolution rates and niche breadth (i.e., specialisation) and covarying traits. No consistent relationships between rates of climatic niche evolution and niche breadth were found in salamanders, and relationships with some climatic variables were positive, indicating higher rates of niche evolution in clades with more generalist niches (Fisher‐Reid et al. 2012). Similar results were found in mammals and damselfish (Cooper et al. 2011; Litsios et al. 2012, but see Satterwhite and Cooper 2015). A recent analysis found significant relationships between rates of climatic niche evolution and niche breadth in less than 20% of ca. 1900 plant and animal species, without a consistent direction of positive or negative relationships (Liu et al. 2020).

The reason for these inconsistent relationships may be a similar one as described above for the relationship between range size and diversification: generalist species (especially those with a wider climatic niche and a larger range size) may have higher rates of niche evolution, but the result of speciation events might be specialised species. Correlative approaches investigating the relationship between niche breadth of extant species and past rates of niche evolution would be driven by recent changes in niche breadth, that is, evolution of specialists into generalists and the other way around (Sexton et al. 2017, see also section 2 Diversification rate). A previous study investigated the relationship between trophic specialisation and environmental niche evolution rates by combining two different approaches: estimating ancestral states of niche breadth states in one analysis, and inferring rates of environmental niche evolution for every branch of the phylogeny in a second analysis (Litsios et al. 2012). However, to our knowledge, no process‐based comparative models exist where rates of niche evolution depend on the niche breadth of the same niche dimension, for example, where environmental niche evolution would depend on environmental niche breadth.

In summary, there are two possible links between high past rates of niche evolution and present‐day extinction risk: the first one predicts decreased extinction risk in lineages with high rates of niche evolution because of their increased evolutionary potential, while the other one predicts increased extinction risk for the same lineages due to specialisation and associated narrow ranges and small population sizes. Initial evidence points towards niche evolution rates being a good predictor for evolutionary potential (Holstad et al. 2024), indicating decreased extinction risk in lineages with high niche evolution rates. However, we found only a single study directly investigating this relationship and more research is necessary. As we found no consistent relationships between rates of niche evolution and niche breadth, niche evolution rates seem to be a poor predictor for current niche breadth/degree of specialisation. It is possible that the proposed negative relationship between niche evolution rates and niche breadth holds only in specific geographic contexts, such as oceanic islands or mountain ranges.

## 5 Synthesis and Recommendations: Can Macroevolutionary Indicators Inform Extinction Risk?

**FIGURE 6:**
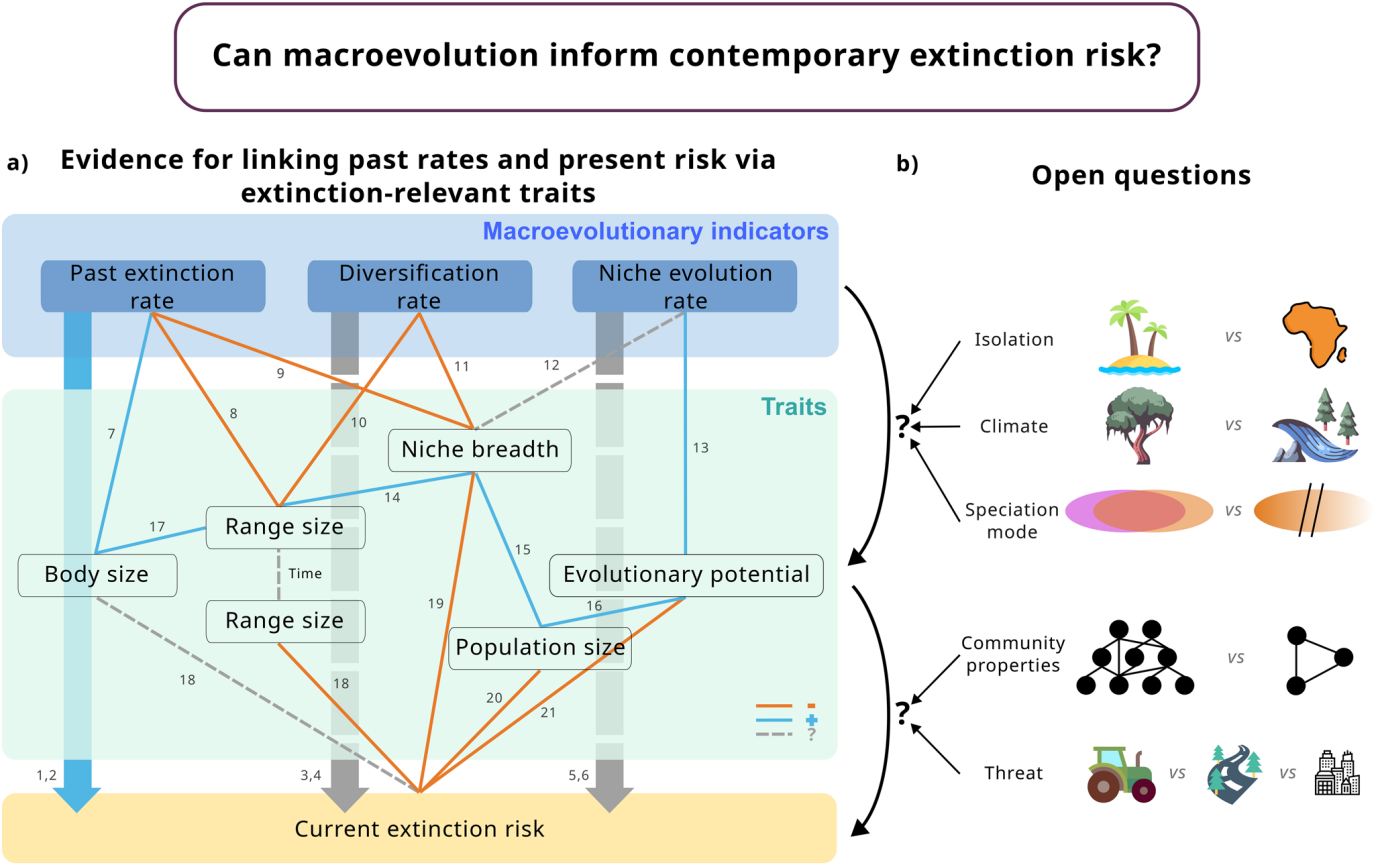
Results from this Synthesis. (a) Evidence for linking past macroevolutionary rates directly to current extinction risk and via extinction‐relevant traits. Red lines indicate negative relationships (e.g., high past extinction rates are linked to small range size), blue lines positive relationships (e.g., high past extinction rates are linked to large body size) and grey dashed lines uncertain relationships. Of all macroevolutionary indicators, only past extinction rates show a clear direct relationship with current extinction risk. (b) The relationships between macroevolutionary indicators and traits of extant species likely depend on different geographic context (e.g., island vs. continent, tropical vs. temperate regions) and speciation mode (sympatric vs. allopatric). In addition, the relationships between species' traits and extinction risk is modified by external factors, such as community properties (species‐rich vs. species‐poor assemblages) and specific threats present in a geographic area (agriculture vs. fragmentation vs. urbanisation). Icons: flaticon.com. Selected references supporting the results: (1) Van Woesik et al. (2012); (2) Roy et al. (2009); (3) Tanentzap et al. (2020); (4) Ye et al. (2024); (5) Lavergne et al. (2013); (6) Rolland et al. (2020); (7) Kemp and Hadly (2015); (8) Condamine et al. (2013); (9) Harnik, Lotze, et al. (2012); Harnik, Simpson, and Payne (2012); (10) Smyčka et al. (2023); (11) Rolland and Salamin (2016); (12) Liu et al. (2020); (13) Holstad et al. (2024); (14) Huang et al. (2021); (15) Gaston (1994); (16) Habel and Schmitt (2012); (17) Sporbert et al. (2021); (18) Chichorro et al. (2019); (19) Chichorro et al. (2022); (20) Purvis, Jones, and Mace (2000); (21) Kardos et al. (2021).

In this article, we have shown a multitude of hypotheses linking macroevolutionary processes to present‐day dynamics (Figure 6a). Integrating past information to inform present‐day processes holds promise, but we also show that underlying mechanistic assumptions need to be explicitly formulated and tested. Overall, current evidence suggests that only past extinction rates are a good predictor of current extinction risk as traits related to past extinctions are also related to current extinction risk. However, the rate at which those traits evolved, specifically niche breadth, can be variable and should be confirmed to be slow for clades of interest. Diversification and niche evolution rates, on the other hand, are currently unlikely to be generally useful predictors because underlying assumptions were only met in specific cases (e.g., restricted geographic space) or hard evidence is only starting to emerge (e.g., linking niche evolution rates to evolutionary potential). In cases where underlying assumptions are met, these indicators can help identify species that are likely at increased extinction risk (inferred from high diversification rates), or at decreased extinction risk due to high evolutionary potential (inferred from high niche evolution rates, but more research is necessary regarding this link).

Future studies using macroevolutionary indicators as indicators of extinction‐relevant traits or as horizon‐scanning tools in an initial assessment of species' intrinsic vulnerability must first verify the validity of the underlying assumptions established in this review (Figures 2, 3 and 5). An initial assessment based on macroevolutionary indicators can then guide in the prioritisation of data collection efforts but conservation decisions will ultimately need to be backed up by additional data (Figure 1). As the use of macroevolutionary indicators is based on a correlative approach and not mechanistic understanding, complex interactions between intrinsic and extrinsic factors related to extinction risk might otherwise not be accurately represented (see also section Limitations and practical considerations). In the following, we highlight the open questions regarding trait‐mediated relationships between macroevolutionary indicators and current extinction risk, indicate future avenues of research and finish with a discussion of general limitations of this approach and practical considerations.

### 5.1 Open Questions and Future Avenues of Research

#### 5.1.1 How Context‐Dependent Are Relationships Between Macroevolutionary Indicators and Species' Traits?

Altogether, there is a pressing need for more studies that investigate the relationships between macroevolutionary rates on traits that are known to be linked to extinction risk, such as range size, niche breadth and evolutionary potential, and how these relationships vary depending on different processes, for example, speciation mode and environmental context (temperate vs. tropical, island vs. continent, Figure 6b). Including past and present geographic and environmental context is therefore a promising avenue of research to better understand in which circumstances our predictions regarding the relationship between macroevolutionary rates and traits hold. Do high diversification or niche evolution rates lead to specialised, small‐ranged species only in geographically restricted contexts? Are low rates of niche evolution in a given clade an indicator of low evolutionary potential, even if that clade occurs in a region that was environmentally stable for a long period of time? In an environmentally stable context, there may not have been any incentive for niches to evolve because past intensity of selection would have been low. Methodological constraints and biases need to be taken into account in these analyses, particularly pertaining to the accuracy of the topology and the dating of the phylogeny for which fossil information is key (Donoghue et al. 1989; Mongiardino Koch et al. 2021).

#### 5.1.2 Which Other Traits Could Be Estimated From Macroevolutionary Indicators?

We see two additional ways of using past information to approximate extinction‐promoting traits. First, past dispersal capacities estimated with historical biogeography models have been linked to invasion success (Gallien et al. 2016, 2019), but we did not find any study investigating the relationship between past biogeographic movements and species' range expansions related to climate change. Especially in high‐latitude environments, long‐distance dispersal has been shown to be an important driver of plant species' range expansions (Alsos et al. 2007, 2015, 2016), and this could prove to be an exciting opportunity for future research. While this approach requires good data of species' extant ranges, it would allow the estimation of an additional trait that is known to be linked to current and future extinction risk but is difficult to quantify: dispersal ability (Trakhtenbrot et al. 2005).

Second, another avenue of research may be investigating patterns in multiple groups to identify ecological interactions that proved to be consistent at the macroevolutionary scale (see also Condamine et al. 2013, 2020). This may help us identify potential dependencies in existing assemblages and anticipate species' interactions in novel assemblages. For instance, by modelling ancestral areas and trait evolution of several clades simultaneously, it is possible to investigate which types of species are likely or unlikely to occur simultaneously (Harmon et al. 2019; Hembry and Weber 2020). Including fossil data in such a setup might provide an additional perspective (e.g., if species with specific traits went extinct more often in the presence (indicating competition) or absence (indicating mutualism) of other species with specific traits). Biotic interactions may prove to be more important than abiotic limits in species' persistence (Cahill et al. 2013; Ockendon et al. 2014), and they can hinder or facilitate species' establishment (Van Kleunen et al. 2018). Indeed, coextinctions have been proposed to dominate future vertebrate losses (Strona and Bradshaw 2022).

#### 5.1.3 How Do the Different Factors Influencing Current Extinction Risk Interact?

Although different extinction‐promoting characteristics interact in complex ways and jointly determine species' extinction risk (Figure 6a), in most studies, these characteristics have been considered separately. For instance, despite niches being multidimensional (including climate, habitat and biotic interaction components such as diet for animals or pollinators for plants), most studies focused on climate niche breadth to determine species' degree of specialisation. Yet, the individual niche dimensions are not necessarily correlated (Carscadden et al. 2020; Emery et al. 2012; Sexton et al. 2017) and specialisation in one dimension might be compensated for by generalism in another (e.g., Litsios et al. 2014). Therefore, it would be valuable for future studies to investigate multiple dimensions simultaneously. To take it a step further, more studies investigating extinction risk in all its complexity are necessary to understand interactions between extinction‐promoting characteristics. For instance, large species are generally at higher extinction risk than small species, but large species also tend to have large ranges (Sporbert et al. 2021), which are generally associated with decreased extinction risk. How do these traits interact with extinction risk in different taxonomic groups and geographical settings?

In addition, realised extinction risk is a combination of intrinsic and extrinsic factors (Figure 6b). On one hand, some clades may be inherently more vulnerable to environmental changes than others due to certain characteristics. On the other hand, different threats impact different areas of the globe, and especially human impact is widespread but not evenly distributed. For instance, the prevalence of risk has been shown to be higher on islands than on continents (Humphreys et al. 2019). While some clades are clustered geographically, others are widely spread across continents, which is why the exposure to threat varies between and within clades. In addition, some ecosystem properties like diversity can impact species' extinction risk. In birds, high local diversity decreases extinction risk of species despite these species holding more extinction‐promoting characteristics (small ranges, large body sizes) on average than species in less diverse assemblages, possibly due to higher levels of ecosystem functioning in diverse assemblages, reduced rates of species' invasion or decreased rates of disease transmission (Weeks et al. 2022). These patterns may be lost in global analyses, and geographically explicit studies are necessary to disentangle the effects of extrinsic and intrinsic factors determining extinction risk.

#### 5.1.4 How Can We Combine Different Scales of Analysis?

Most studies relating diversification rates to current extinction risk were conducted at genus or family level; mostly, they related the proportion of threatened species in a genus or family to genus−/family‐level diversification rates. But how can this relationship at higher taxonomic level inform us about species‐level extinction risk? One way to narrow the taxonomic scale is to measure macroevolutionary indicators within clades below the genus level, thereby capturing within‐genera variation in the traits underlying extinction.

A similar problem has been raised in regards to niche evolution rates. In particular, it has been questioned at which taxonomic level niches and rates of niche evolution should be estimated to inform conservation actions, for example, at species level or below (A. B. Smith et al. 2019). Rates of niche evolution vary depending on the taxonomic level, and adaptations of for example, populations to local environmental conditions have been recorded frequently (e.g., Valladares et al. 2014). Analyses at subspecies level require good data coverage and may be impractical or impossible in many cases, but there is still promise for future investigation (A. B. Smith et al. 2019).

### 5.2 Limitations and Practical Considerations

#### 5.2.1 Accurate Estimation of Macroevolutionary Rates

A key prerequisite for using macroevolutionary rates in a conservation context is the ability to estimate them accurately. However, it is a well‐known problem that estimated rates, especially speciation, extinction and diversification rates, can differ depending on whether they were estimated from the fossil record or from phylogenies (Hagen et al. 2018; Herrera 2017). Even methods that combine the advantages of both approaches (e.g., Mitchell et al. 2019; Silvestro et al. 2018) can still yield discordant rates (e.g., in extinct clades, Cerný et al. 2022) and extinction rates are still difficult to estimate for certain groups of species due to a lack of fossil data. While fossil data and paleontological research remain key for reliable extinction rate estimations, a lot of progress has been made in understanding in which circumstances extinction rates can be estimated from phylogenetic trees (Condamine et al. 2013; Didier et al. 2012, 2017; Morlon et al. 2011, 2022; Quental and Marshall 2010, but see Louca and Pennell 2020).

In the case of niche evolution rates, a problem lies in cladogenetic changes in niche breadth that current methods struggle to reconstruct. Generalist species, particularly if also large‐ranged, may evolve niches faster, yet the speciation process often produces specialists (e.g., Sexton et al. 2017). To accurately represent changes in niche breadth at speciation and improve niche evolution rate estimations, process‐based comparative models would be necessary where rates of niche evolution depend on niche breadth of the same niche dimension, for example, where environmental niche evolution would depend on environmental niche breadth. However, this approach would not fully resolve the issue, as the results would still depend on the specific processes chosen for modelling.

#### 5.2.2 Reliability of Macroevolutionary Rates as Proxies for Extinction‐Promoting Traits

Macroevolutionary rates within a clade are likely to be driven by a combination of intrinsic factors, such as species' traits, and extrinsic factors, including the biotic and abiotic environment (Lawson and Weir 2014; S. A. Smith and Beaulieu 2009). For instance, low niche evolution rates in a clade might reflect low adaptive capacities when the environment changes, leading to a high extinction risk under current climate and land‐use change, but they might also reflect past environmental stability without opportunity or necessity for adaptation, in which case we cannot make any inferences about extinction‐relevant traits (Stigall 2012). As most investigations of the relationship between macroevolutionary rates and traits are correlative and not mechanistic, they need to be interpreted cautiously. Integrating past environmental conditions when estimating macroevolutionary rates could help mitigate this problem.

Evolutionary potential plays a major role in species' responses to environmental changes but it is difficult to measure. While using macroevolutionary rates to make inferences about microevolution (i.e., adaptation to ongoing environmental change) is promising, it is complicated by two major challenges: temporal shifts in evolutionary rates, and varying rate estimates depending on the taxonomic level considered (Rolland et al. 2023; Uyeda et al. 2011). The histories of lineages have been shown to be characterised by long periods of little evolution followed by periods of rapid change (punctuated equilibrium, Gould and Eldredge 1977). However, capturing these rapid pulses is difficult and the average rate of niche evolution will necessarily decrease when measured on a larger time interval. This has an important consequence: the largest evolutionary rates are always measured on shorter timescales, but low evolutionary rates can be inferred at all timescales (Harmon et al. 2021; Uyeda et al. 2011). So a key challenge becomes predicting high potential evolutionary rates that occur in a matter of generations. Holstad et al.'s (2024) study makes an important link by showing that evolutionary potential is positively correlated with niche evolution rates.

#### 5.2.3 Comparability of External Drivers of Extinction Between Past and Present

In the case of using past extinction rates to inform current extinction risk, an additional problem is that drivers of extinction in the past likely differ from drivers of extinction in the present due to human influence, which might be exacerbated in specific settings such as islands (Harnik, Lotze, et al. 2012). This might make past and current extinction incomparable. For instance, direct exploitation by humans is a major threat to many species today without a clear past counterpart which affects species non‐randomly. However, in other instances, the drivers might differ but the effects experienced by organisms may be similar. For example, atmospheric CO_2_ can increase for different reasons (volcanic activity and anthropogenic emissions) but environmental changes that organisms will have to deal with are the same, for example, warming and ocean acidification (Harnik, Lotze, et al. 2012; Payne and Clapham 2012). This is why an in‐depth analysis of the actual traits related to past and current extinction is necessary to contemplate using past extinction rates to inform current extinction risk. As we show in our analysis (section Past extinction rates), the actual traits linked to both past and present extinction are very similar. However, they are also dependent on geography and the type of threat species face (Chichorro et al. 2019), and it has been proposed that the geography of threat may override ecological differences between species and evolutionary history (Lenzner et al. 2020; Verde Arregoitia et al. 2015). While internal drivers of macroevolutionary rates, that is, traits determining population dynamics and vulnerability to change, might be heritable and useful for predicting contemporary or future extinction risk, external drivers might add noise to these relationships that needs to be taken into account.

The problem remains that human influence leads to unprecedented environmental change, which might exceed species' abilities to cope (Pimm et al. 2014). In particular, it is an open question whether species' evolutionary potential can keep up with the speed of current and future climate change. Several studies found that projected climate change is much faster than historical rates of niche evolution (Cang et al. 2016; Jezkova and Wiens 2016; Quintero and Wiens 2013), and the current extinction rate is estimated to be at least 1000 times higher than historical background rates (Lamkin and Miller 2016; Pimm et al. 2014).

#### 5.2.4 Data Availability

The proposal of using macroevolutionary indicators to inform current extinction risk is in parts so tempting because it promises extinction‐relevant information for many species that are currently unassessed due to lack of data. However, this approach still requires data, first and foremost phylogenetic data. While great progress has been made to resolve the tree of life at scale, there is still a lack of phylogenetic data. For instance, the most popular and complete phylogeny of Aves lacks genetic data for 33% of species, and for squamates this rises to 46% of species (Jetz et al. 2012; Tonini et al. 2016). For many other groups, for example, angiosperms or insects, species‐level phylogenies are much less complete. In addition, as we show in this analysis, many underlying assumptions regarding relationships between macroevolutionary rates and traits are context‐dependent and still need to be tested for individual clades or geographic settings. This requires additional data, for example, data on species' niches or life history traits. So how can this approach be valuable when so much additional data is necessary? In many cases, it might be possible to validate assumptions in related clades or similar geographic settings. But more importantly, these phylogenetic approaches still have the potential to approximate traits that are difficult or time‐intensive to measure directly. For instance, while data collection of niche data is labour‐intensive, it is still significantly less so than actually measuring evolutionary potential. When estimating species' vulnerability to current changes, all available data and methodologies should be integrated, leveraging diverse approaches to ensure comprehensive evaluations.

## Author Contributions

L.G. and S.‐S.W. devised the project and the main conceptual ideas; S.‐S.W. conducted the literature review and wrote the original draft; L.G., S.L., F.C.B. and W.L.A. reviewed and edited the manuscript; L.G. and W.L.A. acquired funding.

## Peer Review

The peer review history for this article is available at https://www.webofscience.com/api/gateway/wos/peer‐review/10.1111/ele.70171.

## Data Availability

Data sharing not applicable—no new data generated for this article.
